# A Room Temperature Gas Sensor Based on Sulfonated SWCNTs for the Detection of NO and NO_2_

**DOI:** 10.3390/s19051116

**Published:** 2019-03-05

**Authors:** Eusebiu Ilarian Ionete, Stefan Ionut Spiridon, Bogdan Florian Monea, Elena Stratulat

**Affiliations:** 1 National Research and Development Institute for Cryogenics and Isotopic Technologies—ICSI Rm, Valcea, 240050 Rm. Valcea, Romania; ionut.spiridon@icsi.ro (S.I.S.); bogdan.monea@icsi.ro (B.F.M.); 2 Faculty of Chemistry and Chemical Technology, Department of Chemistry, State University of Moldova, 60 A. Mateevici Street, MD-2009 Chisinau, Moldova; lenuta_stratulat@yahoo.com

**Keywords:** sulfonated CNTs, sensors properties, functional groups, sensing mechanisms

## Abstract

The electrical response of sulfonated single-walled carbon nanotubes (SWCNTs) to NO and NO_2_, for gas sensing applications, at room temperature, is reported in this work. A specific configuration based on SWCNT deposition between double pair configuration gold electrodes, supported on a substrate, was considered for the sensing device; employed characterization technique where FTIR and SEM. The experimental results showed a p-type response of the sulfonated SWCNTs, with decrease in resistance, under exposure to NO gas (40–200 ppb) and NO_2_ (40–200 ppb). Also, the sensor responses to successive exposures at NO_2_ 800 ppb together with investigation of long term stability, at 485 ppb for NO, are reported. The reaction mechanism in case of NO and NO_2_ detection with sulfonated SWCNTs is presented.

## 1. Introduction

The need for gas detection as a safety issue has been recognized even since the beginning of the industrial revolution, when well before the emergence of modern electronic sensors, unconventional alternative solutions, less reliable and less accurate, such as birds (e.g., canaries used as a carbon monoxide and methane detector in coal mines, due to their sensitivity to airborne poisons), have been applied to detect hazardous gases.

Nowadays, technology has evolved and extended to various technical solutions for gas sensing, with the market covering an entire series of commercial products. The most used detection mechanisms include, but are not limited to, electrochemical, catalytic combustion, and recently sensing polymers and semiconductor metal oxides devices [1,2,3,4,5].

With technological progress, need for achieving lower detection thresholds has also increased, partly because of the necessity to confirm the purity of gases for special applications, but more importantly due to the growing concern relative to the global warming and its consequences for future generations. Thus, gas sensors capable of providing reliable information on the state of ambient environment are in strong demand.

Gases as CO, NO, NH_3_ and CH_4_ are frequently found in the polluted atmospheres of our big urban areas, and at different concentration levels are a threat to human health or may constitute a hazard. Nitrogen oxides, mainly NO and NO_2_, together referred to as NOx, are produced from the reaction of nitrogen and oxygen gases in the air during combustion, especially at high temperatures, and represent one of the six most serious pollutants defined by the Environmental Protection Agency (EPA). NOx gas sensors which operate at room temperature [6] also contribute to the formation of fine particles (PM) and ground level ozone, both of which are associated with adverse health effects [7]. Continued or frequent exposure to NO_2_ concentrations higher than the EPA air quality standard (53 ppb) may cause increased incidence of acute respiratory illnesses. For this reason, detection of NO_2_ gas at ppm-level and potentially at ppb-level at room temperature [8] is of tremendous importance.

When discussing continuous air pollution monitoring, the demand for reliable, sensitive and inexpensive analyzers is still to be searched. In the case of NO, an atmospheric pollutant which has an important role in the formation of smog [9], and consequently is a high contributor to human pulmonary diseases [10], the most common available detection techniques are based on chemi-luminescence, photochemical and electrochemical methods [11]. However, these detection solutions lack flexibility, have a relatively low portability and are difficult to use for real-time measurements.

Carbon nanotubes (CNTs) have been proven to be an attractive sensing support substrate to develop sensors for a wide range of applications [12,13,14,15], due to their structure (cylindrical rolled sheets of graphene, presenting a high surface to volume ratio), and mechanical and thermal characteristics. As the building blocks of nanotechnology, CNTs have drawn a lot of attention in the scientific community, with advances in their chemical and physical preparation improving their sensitivity, along with other properties like low power consumption and fast response [16,17,18].

Simple carbon nanotubes are not very sensitive [19] due to the fact that carbon atoms on the CNTs walls are chemically stable due to the aromatic nature of the bonds. Therefore, theoretical and experimental efforts have been devoted to the study and decoration of SWCNTs to provide a response in the form of a change in electrical properties, such as resistance. The process of changing the surface of CNTs by adding useful functional groups, with covalent linkage, onto carbon scaffold of CNTs or at the end caps, is well known as a functionalization process.

Functionalization of the CNTs surface by adding different coatings can enhance the surface reactions or the absorption of specific gases and, as a consequence, enhance and optimize the response of SWCNTs toward a particular gas or mixture of gases [20].

Herein, we propose an innovative solution for high-sensitivity detection of NO and NO_2_, at room temperature, by fabricating a gas sensor with an active substrate based on SWCNTs functionalized with concentrated sulfuric acid (H_2_SO_4_). The assembled gas sensor exhibits highly sensitive NO and NO_2_ sensing. Its performance was tested for a series of mixtures of NO and NO_2_ in N_2_, introduced at different concentrations (from 40 ppb to 800 ppb) highlighting cycle stability and a fast response (255 s for NO in N_2_, 540 s for NO_2_ in N_2_) and recovery time (50 s for NO in N_2_, 420 s for NO_2_ in N_2_) at room temperature. All tests were performed under dry environmental conditions; in our further investigations will consider also the influence of humidity on the measurement performance of the proposed sensing device.

## 2. Experimental Approach

In this work we demonstrate a novel type of NO and NO_2_ sensor based on sulfonated carbon nanotubes, deposited on a skeleton of gold interdigitated electrodes, with the sensing material fabricated through a simple hydrothermal method without any other assistance.

### 2.1. Materials and Methods

**Figure 1:**
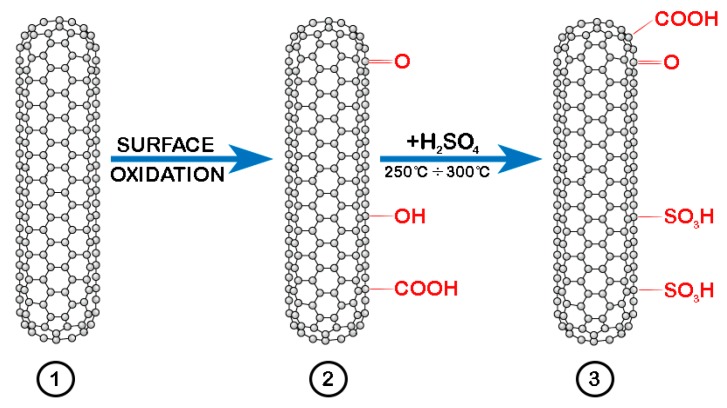
The schematic representation of sulfonated SWCNTs preparation process: 1—SWCNTs purification; 2—oxidation process; 3—sulfonating operation.

The experimental procedure for the preparation of sulfonated SWCNTs is schematically highlighted in Figure 1. To purify the CNTs and remove any remaining particles of catalyst used during the process of nanotubes growth, the SWCNTs (purchased from Sigma-Aldrich Chemie GmbH, Steinheim, Germany) were first mixed in a 1:1 solution of HNO_3_ and HCl, sonicated for 1 h, and then heated up at 60 °C and again sonicated for another 3 hours, resulting thus SWCNT-COOH [19,21]. Next, commercially available sulphuric acid of 98% purity was used on the obtained SWCNT-COOH in order to convert them to SWCNT-SO_3_H. The mixture was sonicated for 30 min and after transferred into a temperature resistant glass container and heated from 60 °C to 300 °C in an electric heater under a protective atmosphere, with a temperature ramp increase of 5 °C/min. Prior to its deposition on a gold interdigitated electrode support, the colloid thus formed was washed with pure water to eliminate any remaining acid, followed by a filtration and drying process. After the drying process, isopropyl alcohol was added to the sulfonated SWCNTs and sonicated for 1 hour, under controlled temperature.

The obtained solution was characterized by using microscopic and spectroscopic methods, namely scanning electron microscopy (SEM) (a NEON® 40EsB Crossbeam System from Carl Zeiss Microscopy GmbH, Jena, Germany, equipped with a thermal Schottky field emission emitter and accelerated Ga ions column), and Fourier Transform Near-InfraRed (FT-NIR) spectroscopy (model Frontier^TM^, produced by PerkinElmer, Waltham, MA, USA, equipped with a diamond Attenuated Total Reflectance -ATR accessory).

Gold interdigitated electrodes deposited on Sitall together with the wire attachment (purchased from Nanospr LLC, Chicago, IL, USA), in a double pair configuration (with the distance between fingers of 50 μm), were used as sensor support substrate. To prepare the gas detection sensor, a little amount of sulfonated SWCNTs solution (approximately 10 μL) was taken and partially dropped onto the Au electrodes, on the ceramic substrate surface. An electrophoresis process, at 10 kV/m, was employed on the CNTs. Applying the electric field to the pair of interdigitated electrodes, SWCNTs suspended into solution are forced by the dielectrophoretic force to move and rotate along the electric field lines.

To set the sensor initial resistance domain in the range of 1 kΩ to 10 kΩ, when the deposited CNTs visually appears to be dry, parts of the solution (almost 1 μL) were dropped on top of the already dry deposited nanotubes. As final manufacturing procedure, a complete drying process of the sensor was performed in an electric oven, under a N_2_ inert atmosphere, for 50 minutes, at 60 °C, with a ramp temperature rise of 2 °C/min.

### 2.2. Gas Sensing Procedure

**Figure 2:**
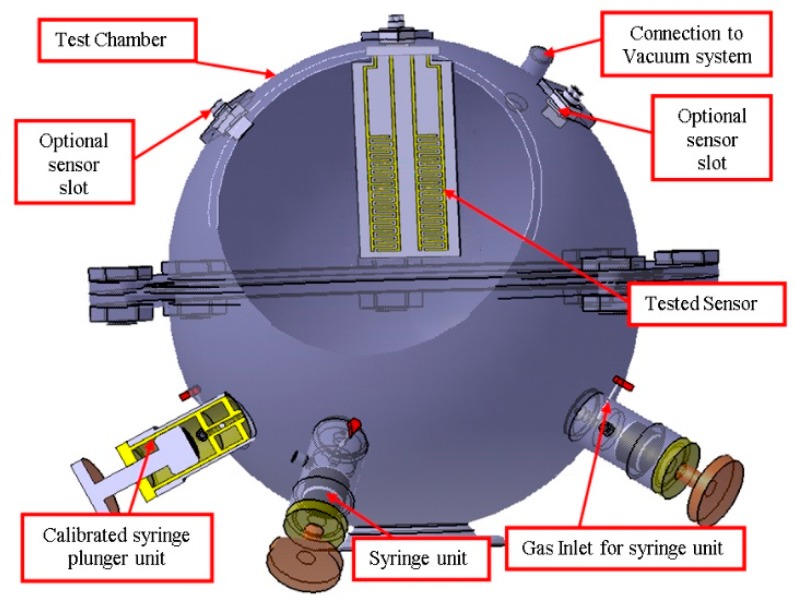
Arrangement of the necessary elements to perform the measurements.

The sensor chip measurement set-up is shown in Figure 2. Dry air, necessary to provide precise control of gas concentration and gas flow was introduced into a test chamber (TC), from a pressurized cylinder. Before starting the experiments, the volume of the TC was carefully determined.

The investigated gases, mixtures of NO and NO_2_ in N_2_, were also introduced from pressurized cylinders with the use of pressure reducers and an introduction device under the form of a special designed calibrated syringe. The values of NO and NO_2_ concentrations in N_2_ gas were previously certified and taken into consideration as such. The CLD chemiluminescence technique was employed to determine the nitrogen monoxide NO, nitrogen dioxide NO_2_, and the total NO + NO_2_, with the minimum detection level of the equipment by the CLD method of 0.5 ppb NO in N_2_, and the measurement uncertainty of 7%.

The manufactured sensor under investigation was inserted inside the TC. After completion the assembly, the TC was evacuated and N_2_ dry gas was introduced to assure a clean environment for the experiments. An E4980 AL 20Hz-1 MHz precision LCR meter (Keysight, Santa Rosa, CA, USA) was used to acquire the electrical signals. This type of acquisition board is capable of achieving a realistic and real time monitoring and analysis of sensor data, useful when no complicated data acquisition system architecture is needed.

## 3. Results and Discussions

### 3.1. Characterization of Functionalized/Sulfonated CNTs

**Figure 3:**
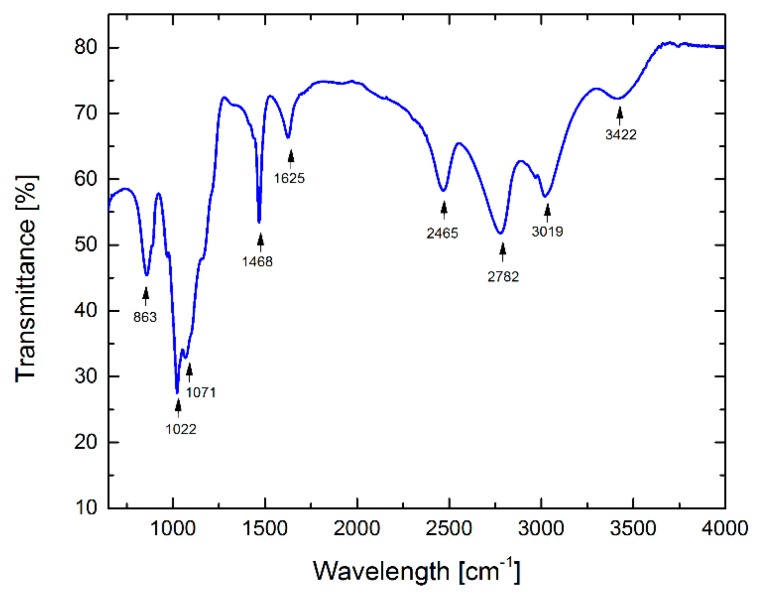
FTIR spectra of as prepared SWCNTs.

A Fourier Transform Near-InfraRed (FT-NIR) spectroscopy analyses, in the domain of 650–4000 cm^−1^, was carried out to identify the functional groups present on the sidewall of the functionalized samples. The FT-NIR spectrum is shown in Figure 3.

**Figure 4:**
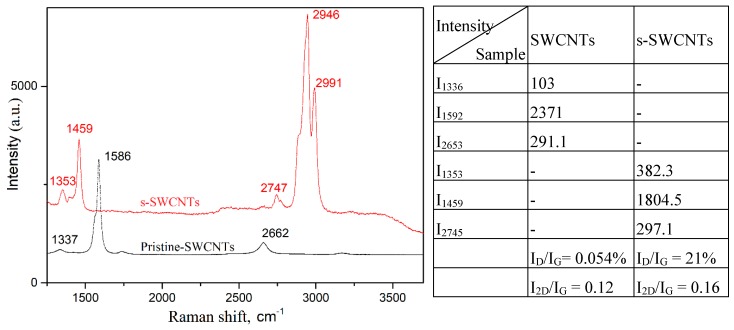
Raman spectra of pristine and sulfonated SWCNTs.

The broad absorption peak at 3422 cm^−1^ is attributed to the presence of phenol hydroxyl groups (–OH) [22]. The three strong peaks at 3019 cm^−1^, 2782 cm^−1^, and 2465 cm^−1^, correspond to symmetric and asymmetric CH vibrations in CH_2_ and CH_3_ groups [23,24]. The absorption band at 1625 cm^−1^ could be attributed to a highly conjugated CO in quinone configuration. The band at 1625 cm^−1^ is characteristic for the C=C bonds in CNTs, and 1468 cm^−1^ is due to the C-O carboxylate anion stretching mode [25]. The peaks of 1071 cm^−1^ and 1022 cm^−1^ correspond to the aromatic C–C stretching vibration mode. The high peak at 863 cm^−1^ can be assigned to O-S-C stretching groups confirming that the carbon nanotube’s surface was successfully sulfonated by sulfuric acid. FTIR characterization of the nanotube samples after functionalization confirms that the SWCNTs were covered with sulfonic acid groups. Also, by using Raman spectroscopy we verified the covalent functionalization of the SWCNTs, before and after the acid treatment procedure (Figure 4). The peak at 1592 cm^−1^ is blue shifted at 1459 cm^−1^, while the peak at 2652 cm^−1^ is red shifted at 2745 cm^−1^. This indicated that a process of functionalization has occurred which induced defects in the SWCNTs. The efficiency of the sulfonated process can be estimated from the intensity ratio at different wavenumbers. The ratio between bands D and G in SWCNTs indicates an ordered structure, with few defects. This ratio increases to 21% in the sulfonated SWCNT (s-SWCNT).

Our results are in good agreement with the literature, which states that the increase in ratio of intensities is produced by the functionalization of the sidewalls of the SWCNTs [26]. The ratio between bands 2D and G is very close between the two samples and shows the efficiency of the functionalization process (1.33%).

### 3.2. Structural Characterization

**Figure 5:**
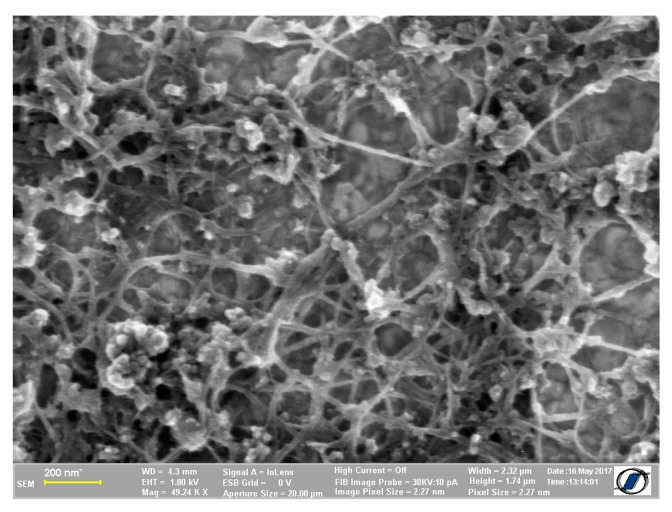
SEM image of the sulfonated SWCNTs.

Figure 5 show scanning electron microscopy (SEM) image of the sulfonated SWCNTs on sensor substrate. It can be seen that, on the surface, the nanotubes are tangled with each other in a random network.

### 3.3. Gas Sensing Characterization

Prior to sensing experiments, the TC atmosphere was cleaned by evacuating it. Next, a current of dry N_2_ gas was introduced in the chamber for minimum 10 minutes. This procedure was repeated after each sensor exposure to gas.

#### 3.3.1. Performance of sulfonated-SWCNTs nanocomposite in detecting NO in N_2_

**Figure 6:**
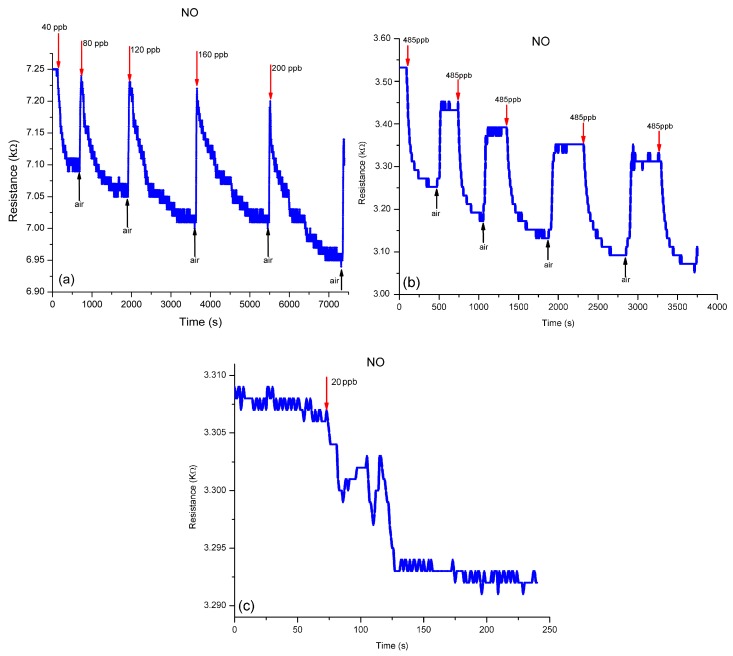
Sensing to different NO exposure: (**a**) sensor response toward variation of different NO in N_2_ concentrations; (**b**) sensor repeatability to exposures at a concentration of 485 ppb NO in N_2_; (**c**) sensor response on exposure at 20 ppb NO in N_2_.

The behavior of sulfonated-SWCNTs nanocomposite when exposed to different concentrations of NO in N_2_, namely 40, 80, 120, 160, 200, 485 and 800 ppb, at different time intervals, is highlighted in Figure 6. Sensor response is presented (Figure 6a) as resistance variations at different NO exposures. A gradually increase, in the amplitude magnitude, of the sensor response is observed, even if the value of resistance decreases with the increase of gas concentration.

A successive introduction in the TC of the same NO gas concentration (485 ppb) was performed to measure the sensor repeatability. At gas exposure, the sensor response time, shown in Figure 6b, is between 255 s up to 370 s, with the regeneration cycles, and after air exposure, from 50 s to 90 s. From the obtained characteristic trace (Figure 6c) it can be seen that intimate changes have taken place in the sensor sensible material. Also, the presence of electrical noise, in the form of low resistance variations, can be visualized.

By measuring the sensor performance at an exposure of 20 ppb NO in N_2_, we intend not to establish a detection limit, but to study if the sensor can exhibit a response to a very short stimulus, at a very low concentration, even the response was not on the sensor general tendency.

**Figure 7:**
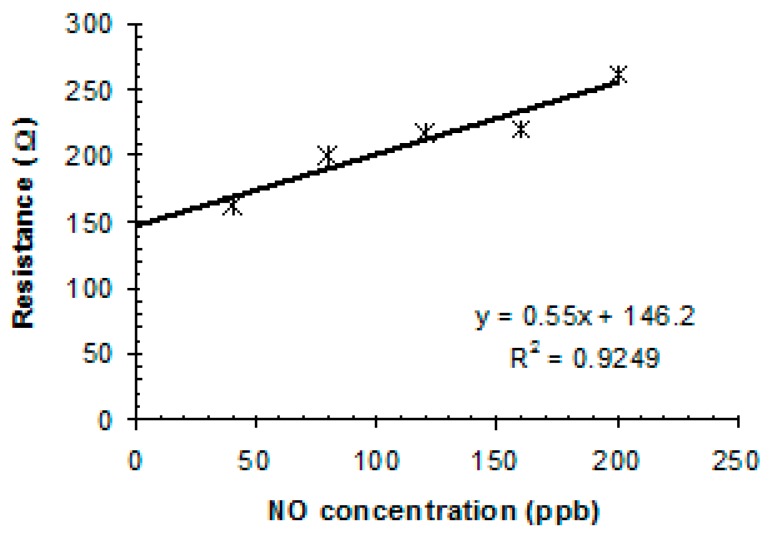
Linear fitting of sensor response toward ppb-level of NO gas.

To explore the sensor behavior in relation with NO gas concentration, investigations where conducted by exposing the sensor in the domain of 40–200 ppb, as shown in Figure 7. The experimental fitting curve is rather linear in this domain, with a good approximation.

#### 3.3.2. Performance of Sulfonated-SWCNTs Nanocomposite in Detecting NO_2_ in N_2_

**Figure 8:**
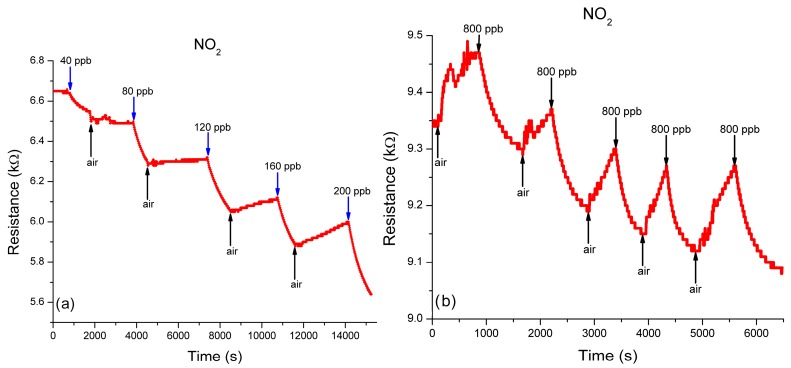

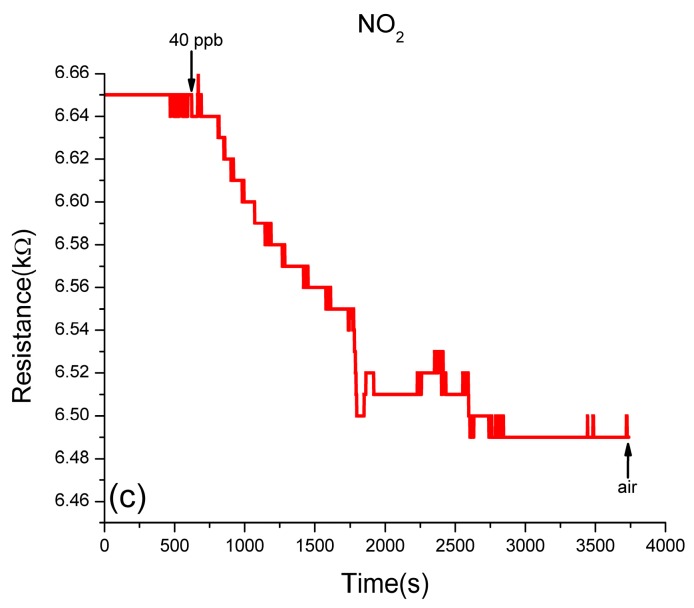
Sensing to different NO_2_ exposure: (**a**) sensor response to exposure at different concentration of NO_2_ in N_2_, starting from 40 ppb up to 200 ppb; (**b**) sensor repeatability to exposure at a concentration of 800ppb NO_2_ in N_2_; (**c**) sensor response to an exposure at a concentration of 40 ppb NO_2_ in N_2_.

As a second step, the sensor platform was exposed to different mixtures of NO_2_ in N_2_, starting from 40 ppb to 200 ppb, at different time intervals as shown in Figure 8, under the same experimental procedure as for NO.

Figure 8a shows the sensor characteristics obtained when the concentration of the NO_2_ in N_2_ introduced in the TC is gradually increased from 40 ppb up to 200 ppb, with an increment of 40 ppb per step. When exposed to a single concentration of 800 ppb NO_2_ in N_2_, the sensor response time is between 540 s to 830 s, while the regeneration cycles, after air exposure, is between 420 s up to 700 s (Figure 8b).

**Figure 9:**
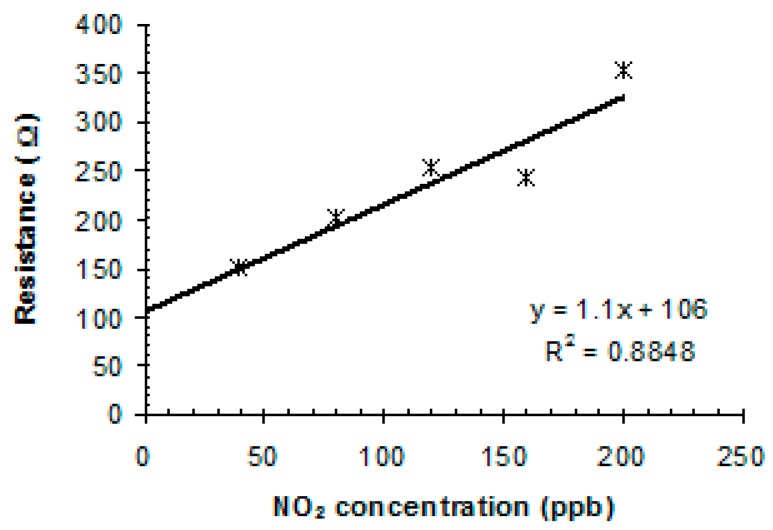
Linear fitting of sensor response toward ppb-level of NO_2_ gas.

Similar for the NO, in the case of NO_2_, the sensor response was tested in the same range of concentrations, from 40 ppb (Figure 8c) to 800 ppb (Figure 8b). The experimental fitting curve, shown in Figure 9, is also linear in this domain, presenting the same good approximation.

It can be concluded from Figure 8 and Figure 9 that the actual sensitivity of the sensing platform is lower and, judging by the fitting curve allure, the sensor sensitivity can also be improved by changing the sensor initial resistance.

#### 3.3.3. Recovery after exposure to NO and NO_2_

When a sensor is integrated into a functional instrument, such as a transducer, it is a natural desire for the users to have a fast and full recoverable device, after the input stimulus is gone, e.g., after each cycle of exposure to gas is over. According to the intensity of stimulus, the recovery process must be also very fast (e.g., a few seconds), this sensor characteristic being of a great importance and sometimes a challenge.

The process of thermal recovery (heating of the sensor sensible substrate) is a well-known and used method [27]. It does have the limitation in the form of the need for an electrical heater integrated on the same chip as the sensor support. Another limitation is related to the heater power consumption that must be added to the device total power consumption.

In the case of our device the recovery process was studied without heather, just by natural exposure to dry gas. The recovery process was in a natural fashion, taking advantage of the simplicity of the process and also of the deposition layout of the sensible substrate, under the form of a special architecture of bridges of nanotubes between gold interdigitated electrodes. The sensor response increased gradually, in amplitude, according to the increase of gases concentration, so the regeneration time has evolved, has increased in the same manner.

#### 3.3.4. Exposure to H_2_ and CH_4_

**Figure 10:**
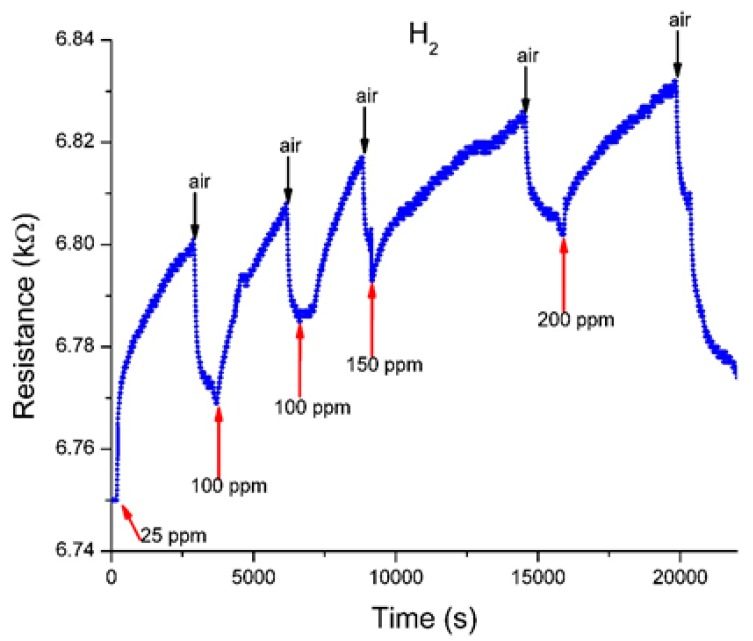
The sensor response to exposure at different concentration of H_2_ in N_2_.

**Figure 11:**
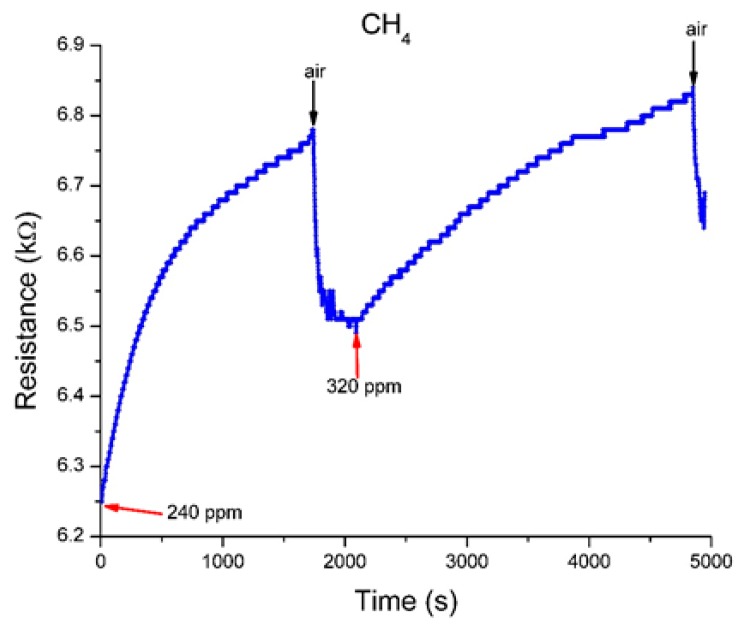
The sensor response to exposure at different concentration of CH_4_ in N_2_.

An alternative sensing response performances of the sensor was also tested, upon exposure at two other different gases, hydrogen H_2_ and CH_4_ (Figure 10 and Figure 11).

For the proposed sensor, the sensing repeatability was tested for five consecutive sensing cycles, starting from 25 ppm up to 200 ppm in case of H_2_, and at 240 ppm and 320 ppm for CH_4_. Also, the recovery behavior was registered. The measured increase in the resistance values of the sensing substrate can be explained as follows: H_2_ and CH_4_ are electron donor gases. When the sensing substrate is exposed to H_2_ or CH_4_ molecules electrons will be transferred to s-SWCNTs, thus decreasing the number of conducting holes and increasing the resistance of sensing substrate.

The sensors present comparable response times (of thousands of seconds), faster recovery, but became sensitive at values larger than in the case of NO or NO_2_ (Table 1).

**Figure 12:**
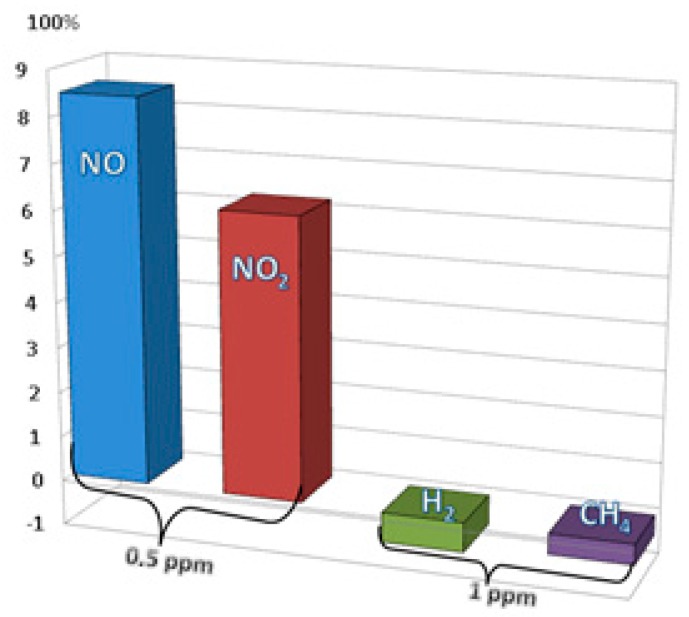
The sensor response to exposure at different gases.

It can be concluded that one gas (CH_4_) has a faster response time and lower sensor regeneration time than other. Selectivity of the sensing film was also examined with the exposure of 1 ppm mixture in N_2_ of H_2_ and CH_4_ inside the TC. The registered sensitivity was found to be very low compared with that for NO and NO_2_ (Figure 12), the sensor responded but the results were visibly insignificantly.

Hence, these graphs support the statement that sulfonated SWCNTs are good for the detection of NO and NO_2_, at very low concentration, at room temperature.

### 3.4. Mechanism of Detection

The reaction mechanism in case of NO and NO_2_ detection with sulfonated SWCNTs is presented here, considering that several steps are foreseen in the reaction. When the gas reaches the sensible surface of SWCNTs covered with main acidic functionalities, such as sulfonic group –SO_3_H, carbonyl –C=O and hydroxide group –OH, a complex adsorption mechanism, physical [28] and/or chemical [29,30,31], will start.

The observed recovery time, from tens of seconds (for NO) up to hundreds of seconds (for NO_2_), lead us to conclude that chemisorption is the main detection mechanism [32]. The sulfonic acid group –SO_3_H is the main investigated group for the proposed sensors, therefore the next discussions are focused on it. Firstly, on the surface of functionalized SWCNT with sulfonic groups –SO_3_H, the positive charges are transferred from the SWCNTs films to the surface, where the formed chemical component (SO_3_H) is attached. SWCNTs behave like p-type materials, meaning that a number of holes have already been present inside the valence band, due to electron withdrawing by oxygen molecules adsorbed on the CNTs surface. Thus, by attaching sulfonic group –SO_3_H, the SWCNTs films became more hole-doped. The electron rich sites, as the lone pair of electrons of S or O atoms in sulfonic group –SO_3_H, tend to adsorb the electron-withdrawing molecule, in our case NO_2_ or NO, since both gases more usually act as oxidizing agents. Concluding, the NO_2_ and NO behave as acceptors to induce holes charge carriers, which are responsible for the sensor response.

When NO_2_ and NO gas appear around SWCNTs functionalities, the electron charge is extracted and transferred to the surrounding gas; SWCNTs will donate electrons and will end up with more holes in the valence band, making the SWCNTs films more conductive. This result is consistent with the resistance change of SWCNTs layer measured in the experimental results.

With the introduction of air, the regeneration process starts and gas molecules are easily desorbed from the film surface due to the weak combination of the gas molecule and the lone-pair electrons in –SO_3_H [33].

## 4. Conclusions

Sulfonated SWCNTs were prepared by treatment of pure SWCNT with sulphuric acid (H_2_SO_4_), from 60 °C up to elevated temperatures (300 °C). During these treatments, different chemical groups were attached on the surface of SWCNTs. The resulting functionalized SWCNTs were dispersed in isopropyl alcohol and a small quantity was electrodeposited on interdigitized electrodes.

We have successfully demonstrated that a sensor based on sulfonated SWCNTs, fabricated by drop-cast method followed by dielectrophoretic alignment, is feasible to be fabricated. The obtained sensor shows a high sensitivity (exhibiting an enhanced response) toward NO and NO_2_ in nitrogen gas with a detection range of 40–800 ppb. The response time was determined to be 255 s in the case of NO and 540 s in the case of NO_2_, at room temperature. The recovery time was determined to be relatively faster than detection time, 50 s for NO and 420 s for NO_2_.

The employed manufacturing approach seems to have some deficiencies and limitations, related to device surface quality and performance. It is difficult to obtain a measuring device preset for multiple specific ranges of gas concentrations. Two similar sensor structures manufactured at the same time and under the same conditions can have a high degree of value dispersion.

In the evolution of the resistance values (sensor response) subjected to 40 ppb NO in N_2_, taken during experiments, fluctuations, tremors, which can be attributed to the vibrations and reassessments of the substrate sensitive to the support surface between the gold electrodes are observed. This is one of the limitations of the deposition technique used—drop casting—which does not ensure a secure fixation of carbon nanotubes on contact surfaces.

Future work will focus in investigating the interaction of NO and NO_2_ gas molecules with different functional groups introduced on the surface of different types of CNTs, the resulting CNTs network differences as sensitivity and selectivity and modalities to improve the sensor recovery time. It is a necessity to improve the assembly techniques for sensor manufacture in order to obtain appropriate network resistances and to minimize the random value dispersion and to set specific domains.

The presented sensor, due to its low power consumption and portability, can be integrated into devices, such a future nanoelectronic nose, useful in stringent applications like wireless environmental monitoring.

With the increase in human population and growing concerns about the environmental pollution the easy detection, at lower levels, of different pollutants has gained critical importance.

## Figures and Tables

**Table 1:** Sensor response and regeneration for different H_2_ and CH_4_ concentrations.

Gas Process	H_2_ (ppm)					CH_4_ (ppm)	
	25	100	125	150	200	240	320
Response	2579	2347	1480	5233	3897	1694	2693
Regeneration	889	822	521	1352	2483	352	489
